# Duodenum-preserving pancreatic head resection compared to pancreaticoduodenectomy: A systematic review and network meta-analysis of surgical outcomes

**DOI:** 10.3389/fsurg.2023.1107613

**Published:** 2023-03-29

**Authors:** Shixiang Guo, Qiang Zhou, Jiali Yang, Junyu Tao, Junfeng Zhang, Huaizhi Wang

**Affiliations:** ^1^ Institute of Hepatopancreatobiliary Surgery, Chongqing General Hospital, Chongqing, China; ^2^ Chongqing School, University of Chinese Academy of Sciences, Chongqing, China

**Keywords:** duodenum-preserving pancreatic head resection, organ sparing, chronic pancreatitis, pancreatic head benign lesion, pancreatic head low-malignant lesion

## Abstract

**Objectives:**

In this systemic review and network meta-analysis, we investigated pancreaticoduodenectomy (PD), pylorus-preserving pancreaticoduodenectomy (PPPD), and different modifications of duodenum-preserving pancreatic head resection (DPPHR) to evaluate the efficacy of different surgical procedures.

**Methods:**

A systemic search of six databases was conducted to identify studies comparing PD, PPPD, and DPPHR for treating pancreatic head benign and low-grade malignant lesions. Meta-analyses and network meta-analyses were performed to compare different surgical procedures.

**Results:**

A total of 44 studies were enrolled in the final synthesis. Three categories of a total of 29 indexes were investigated. The DPPHR group had better working ability, physical status, less loss of body weight, and less postoperative discomfort than the Whipple group, while both groups had no differences in quality of life (QoL), pain scale scores, and other 11 indexes. Network meta-analysis of a single procedure found that DPPHR had a larger probability of best performance in seven of eight analyzed indexes than PD or PPPD.

**Conclusion:**

DPPHR and PD/PPPD have equal effects on improving QoL and pain relief, while PD/PPPD has more severe symptoms and more complications after surgery. PD, PPPD, and DPPHR procedures exhibit different strengths in treating pancreatic head benign and low-grade malignant lesions.

**Systematic Review Registration:**

https://www.crd.york.ac.uk/prospero/, identifier: CRD42022342427.

## 1. Introduction

As a part of the pancreas, the pancreatic head has exocrine and endocrine functions. Numerous benign and low-grade malignant lesions involving the pancreatic head require surgical intervention (1). Chronic pancreatitis (CP) or CP with pancreatolithiasis comprises a large part of a pancreatic head benign lesion, and it causes parenchymal or intraductal calcifications, pancreatic fibrosis, and exocrine and endocrine pancreatic insufficiency. With the progression of CP, lots of patients often need to undergo surgical intervention due to severe abdominal pain, reduced quality of life (QoL), occlusion of the portal vein, obstruction of the duodenum, common bile duct (CBD), main pancreatic duct, and so on (2, 3). Low-grade malignant lesions in the pancreatic head generally include intraductal mucinous neoplasms, solid pseudopapillary neoplasm, neuroendocrine tumor, and serous/mucinous cystadenoma, while these have a low incidence of metastasis and good prognosis with surgical resection (4, 5).

Pancreaticoduodenectomy (PD) and pylorus-reserving pancreaticoduodenectomy (PPPD) are traditional surgical approaches for treating lesions involving the pancreatic head and periampullary regions (6). Although PD and PPPD completely remove the primary lesions, their disadvantages remain significant, including great changes to the digestive tract, procedural complexity, and more complications based on anatomic and physiologic complexity (7).

Many surgeons designed and performed various novel procedures to further improve the efficacy and reduce the complications of surgical procedures. Beger et al. (8) reported a new surgical strategy for benign and low-grade malignant lesions. Since then, duodenum-preserving pancreatic head resection (DPPHR) and its modifications have been employed in treating pancreatic head lesions (1, 7, 9). These surgical procedures can potentially preserve the adjacent organs and exocrine/endocrine functions of the pancreas while reducing complications and mortality (10). Diener et al. stressed that DPPHR procedures had superiority over PD in hospital stay, weight gain, exocrine insufficiency, QoL, and other postoperative outcome parameters (11). DPPHR had better short-term results and a longer survival time than PD (12). The clinical evidence may be the verification of DPPHR advantages. Previous studies have compared different DPPHR procedures (13–15). However, they explored only a few parameters. The advantages and disadvantages of different DPPHR procedures need to be further investigated. Also, there is no study comparing different DPPHR procedures and systemically direct/indirect comparing PD/PPPD/DPPHR simultaneously. In this study, we developed meta-analyses and network meta-analyses to compare different surgical procedures directly/indirectly for pancreatic head benign/low-grade malignant lesions in terms of postoperative outcomes.

## 2. Methods

### 2.1. Search strategies and selection criteria

Because our study was a systemic review of the published literature, ethical approval, and written consent were not required. Before the literature search, we determined to review PICO: “P” for patients with pancreatic head lesions, “I” for operations involving the pancreatic head, “C” for different surgical procedures, and “O” for multiple postoperative outcomes. A computerized search was performed using databases PubMed, Web of Science, Embase, Cochrane Library, CNKI, and WANFANG DATA in August 2021 with the combination of keywords “benign lesion or low-grade malignant lesion,” “chronic pancreatitis,” “pancreatolithiasis,” “duodenum preserving,” “organ sparing,” “organ preserving,” and “pancreatic head resection.” Results were limited to human studies and English papers but not meeting abstracts, case reports, and reviews. Articles and meta-analyses were included. Basic information including title, authors, abstract, and publication information was exported for primary review.

Two authors reviewed/selected the literature, extracted data, and assessed the bias of enrolled literature independently. We kept papers closely related to the subject of this research for following detailed reading after the first review of the title and abstract. Studies in non-English writing, without access to the full paper, having insufficient statistics, or suspicious of redundant publication were excluded. Resting studies were included in quality assessment and data extraction.

### 2.2. Data extraction and assessment for risk of bias

After fully evaluating enrolled papers, we extracted the data related to postoperative function assessment, postoperative symptom investigation, surgical and hospitalization parameters, and baseline of studies. The specific indexes are listed in the following tables. Continuous data were presented in means with standard deviations (±SDs) or medians with ranges/interquartile ranges (ranges/IQRs); dichotomous data were expressed in numbers. All data were extracted for the same trial reported in different follow-up periods, and only the latest data were used for the overall comparison.

The risk of bias in randomized controlled studies (RCTs) was evaluated according to the *Cochrane Handbook for Systematic Reviews of Interventions* (16). The evaluation of observational studies was scored based on the Newcastle–Ottawa scale (NOS) (17). Low-quality literature was excluded based on risk assessment. If there were discordance in literature selection, data extraction, and bias assessment between reviewers, we rechecked the course or consulted another author until an agreement was reached.

### 2.3. Data synthesis and analysis

We analyzed dichotomous data (e.g., morbidity, pancreatic fistula) as a risk ratio (RR) with a 95% confidence interval (CI) and continuous data as a mean difference (MD) with 95% CI when the outcome was reported or converted to the same units (e.g., hospital stay) or as a standardized mean difference (SMD) with 95% CI when scales were used (e.g., QoL). We converted the medians (IQRs/ranges) into the means (±SDs) through a method published by Luo et al. (18).

We assessed heterogeneity using the *I*^2^ tests to evaluate the overlap of 95% CIs. The fixed-effects model was adopted for low heterogeneity (*I*^2^ ≤ 50%), the random-effects model was adopted for greater heterogeneity (*I*^2^ > 75%), and subgroup analysis was explored. We developed a funnel plot to explore potential publication biases if the subgroup included more than five trials.

Our research is reported in line with PRISMA (19). All the syntheses and analyses were conducted in Revman 5.4 and STATA 14 software. A *p*-value less than 0.05 could be of statistical significance. This work has been registered in PROSPERO (ID: CRD42022342427).

## 3. Results

### 3.1. Search results

**Figure 1:**
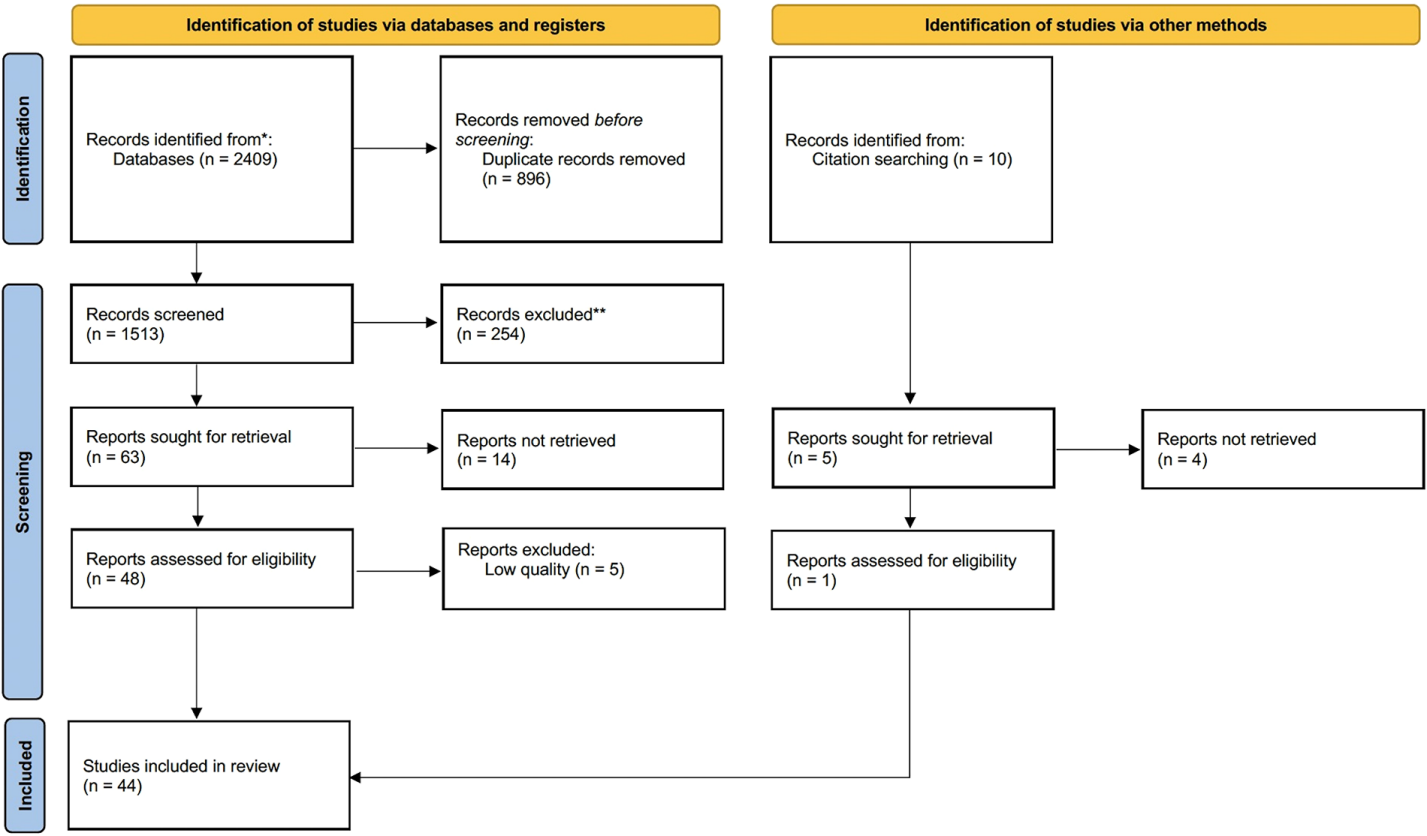
Flowchart of literature screening.

Following our search strategy, 2,409 pieces of literature (809 PubMed studies, 923 Web of Science studies, 77 Embase studies, 60 Cochrane Library studies, 234 CNKI studies, 306 WANFANG DATA studies, and 10 records identified through other sources) were exported. Only 44 studies (Supplementary List S1) were enrolled in the final data synthesis after full paper reading and quality assessment by two authors. Figure 1 shows the flowchart of this study.

### 3.2. Included studies

Characteristics of the enrolled non-randomized studies are shown in Table 1, and those of RCTs and long-term follow-ups of RCT are presented in Table 2. This study included seven RCTs, six long-term follow-ups of RCT, 30 retrospective and prospective cohort studies, and one long-term follow-up of a prospective study. All of the studies included 3,471 patients, of which 14 studies involving 350 patients investigated benign or low-grade malignant lesions and 30 studies (including all RCTs and long-term follow-up of RCTs) investigated CP. However, only five retrospective studies and one RCT had ≥50 patients per subgroup. No RCTs were excluded from our study. NOS scores of 8, 7, and 6 were assigned to 13, 13, and 5 nonrandomized studies, respectively. The gender distribution, age range, and follow-up duration varied among studies. Studies were excluded with reasons and presented in the flowchart (Supplementary Figure S1).

**Table 1:** Characteristics of enrolled non-randomized studies.

Studies	Study design	Diagnosis	Study period	Treatment group	Patient number	Sex (M/F)	Age	Follow-up duration (month)	NOS score
Keck (2010)	Retrospective	Chronic pancreatitis	1996–2007	Frey	50	39/11	45 (27–78)	43 (range 8–126)	7
				Beger	42	36/6	41 (30–62)	62 (range 6–137)	
Bellon (2019)	Retrospective	Chronic pancreatitis	–	DPPHR (Hamburg)	406	304	49.2 ± 11.1	119 (mean)	7
				DPPHR (Beger/Frey)	79	61	43.6 ± 10.5	126 (mean)	
Belina (2005)	Retrospective	Chronic pancreatitis	1998/4–2002/12	Frey	56	55/1	45 (23–71)	39 (median)	7
				PD/PPPD	48	39/9	48 (29–71)		
Busquets (2010)	Retrospective	Benign or low-grade malignant lesion	1989–2006	DPPHR (DPPHR/EN/UC)	24	12/12	51 ± 13	–	6
				PD/PPPD	41	33/8	46 ± 14		
McClaine (2009)	Retrospective	Chronic pancreatitis	1999/9–2006/8	PD	59	26/33	46.8 ± 11.1	47 ± 20	8
				DPPHR (Beger/Frey/Berne)	22	8/14	44.9 ± 11.1	14 ± 8	
Sun (2020)	Retrospective	Benign or low-grade malignant lesion	2014/1–2018/12	DPPHR (Beger/Frey/Berne)	29	15/14	45.48 ± 13.05	≥12	7
				PD	57	28/29	50.89 ± 15.31		
Witzigmann (2003)	Prospective	Chronic pancreatitis	1996/1–2000/12	PD	32	25/7	47 ± 12	9–12	8
				Beger	38	28/10	42 ± 10		
				PD	32	25/7	47 ± 12	18–24	
				Beger	38	28/10	42 ± 10		
Zheng (2012)	Retrospective	Chronic pancreatitis	2004/1–2009/6	PD/PPPD	57	51/6	45.6 ± 9.7	34.8 (range 6–36)	8
				DPPHR (Beger/Frey/Berne)	66	51/15	46.0 ± 8.8		
Jiang (2018)	Retrospective	Benign or low-grade malignant lesion	2010/1–2016/12	RDPPHR (mBeger)	34	8/26	47.0 ± 14.5	32 (range 3–79)	8
				RPD	34	13/21	47.4 ± 14.7		
Li (2017)	Retrospective	Benign or low-grade malignant lesion	2008/2–2014/11	PHRSD	20	6/14	49.5 ± 4.6	47 (median)	8
				PPPD	42	23/19	49.8 ± 2.3	65 (median)	
				PD	92	47/45	48.4 ± 1.7	60 (median)	
Ahn (2003)	Retrospective	Benign, low-grade malignant lesion	1995/1–2001/9	DPPHRt	8	4/4	range 26–62	–	6
				PHRSD	7	3/4	range 34–66		
Horiguchi (2010)	Retrospective	benign or low-grade malignant lesion	–	PPPD	19	11/8	67.2 ± 7.2	2–216	6
				DPPHR (mDPPHRp)	21	11/10	59.5 ± 13.6		
Kelemen (2002)	Retrospective	Chronic pancreatitis	1991–1998	Beger	32	26/6	45.3 (36–64)	41.5 (range 3–89)	7
				Frey	13	13/0	45.9 (36–58)	20.6 (range 3–46)	
				PPPD	21	19/2	48.2 (31–70)	31.1 (range 6–61)	
Pedrazzoli (2011)	Retrospective	Benign and premalignant lesion	1991/1–2008/12	DPPHR (mDPPHRt/p)	27	10/17	54 (33–74)	85 (range 3.3–204.5)	7
				PPPD	37	23/14	61 (45–84)	60 (range 24–216)	
Chiang (2007)	Retrospective	Chronic pancreatitis	1996/1–2003/12	PD	17	16/1	40.4 ± 15.1	6	7
				Frey	25	20/5	39.7 ± 11.6		
Chen (2020)	Retrospective	Benign or low-grade malignant lesion	2016/1–2019/12	LDPPHR (DPPHRt)	15	4/11	54.7 ± 13.9	3	7
				LPD	39	24/15	61.5 ± 11.9		
Nakao (2007)	Retrospective, multicenter	Intraductal mucinous neoplasms	1996/3–2006/3	PHRSD	35	15/20	65.1 ± 9.0	42.8 ± 27.7	8
				PPPD	32	14/18	61.7 ± 8.8	76.2 ± 48.9	
Umemoto (2019)	Retrospective	Low-grade malignant lesion	2009–2017	DPPHR (DPPHRt)	13	2/11	43 (13–78)	47 (range 14–109)	8
				PD	14	6/8	60.5 (40–83)		
Benzing (2018)	Retrospective	Chronic pancreatitis	1995–2013	PD	79	68/11	50 (29–80)	86.4 (range 0–216)	7
				DPPHR (Beger/Frey/Berne)	52	45/7	43 (17–61)	104.4 (range 12–180)	
Hildebrand (2010)	Retrospective	Chronic pancreatitis	2000/3–2005/4	Frey	39	30/9	46.6 ± 9.1	50 (median)	6
				PD	12	10/2	54.1 ± 9.7		
Sun (2014)	Retrospective	Chronic pancreatitis	2008/9–2014/4	Beger	24	21/3	40 ± 3	36.3 (mean)	8
				Berne	22	20/2	40 ± 3		
Liu JZ (2012)	Retrospective	Chronic pancreatitis	2004/1–2010/12	PD	37	19/18	47.3 ± 12.2	42 ± 19	8
				Beger	22	12/10	45.7 ± 10.8	20 ± 9	
Liu CJ (2012)	Retrospective	Chronic pancreatitis	2005/1–2011/5	DPPHR	24	9/15	40.5 ± 15.9	–	7
				PD	40	16/24	41.2 ± 14.8		
Liu (2019)	Retrospective	Chronic pancreatitis	2014/3–2017/3	Frey	62	43/19	48.26 ± 11.50	12	8
				Beger	62	41/21	48.05 ± 12.33		
Gao (2019)	Retrospective	Chronic pancreatitis	2011/1–2012/6	Frey	31	21/10	45.7 ± 8.9	45 (range 20–70)	6
				PD	10	8/2	53.1 ± 9.6		
Du (2011)	Retrospective	Chronic pancreatitis	1999/1–2009/6	Frey	32	20/12	46.19 ± 7.06	57 (range 6–114)	8
				Beger	38	26/12	48.05 ± 7.20		
Cheng (2020)	Retrospective	Benign or low-grade malignant lesion	2014/1–2019/12	DPPHR (Beger/Frey/Berne)	35	19/16	44 ± 15	20 (range 12–60)	7
				PD	67	32/35	52 ± 15		
Wang (2018)	Retrospective	Benign or low-grade malignant lesion	2013/9–2014/9	Takada (DPPHRt)	6	4/2	37–72	30–41	7
				PD	6	4/2	44–68		
Tsuchikawa (2013)	Retrospective	Low-grade malignant lesion	1994–2011	DPPHR (Berne)	8	5/3	58 (23–70)	51 (median)	7
				DPPHR (mDPPHRt)	13	3/10	63 (29–77)		
Möbius (2007)/Witzigmann (2003)	Prospective	Chronic pancreatitis	–	PD	24	–	–	63.5 (range 6–56)	8
				Beger	29	–	–		
Fujii (2011)	Retrospective	Benign or low-grade malignant lesion	1991/7–2009/12	PHRSD	77	45/32	61.0 (26–84)	45.6 (median)	8
				PPPD	55	35/20	62.4 (20–82)	42.5 (median)	

Conventional pancreatoduodenectomy or Whipple procedure as PD, pylorus-preserving PD as PPPD, duodenum-preserving pancreatic head resection as DPPHR, DPPHR procedure of pancreatic head resection with segmental duodenectomy as PHRSD, modifications of DPPHR procedures with total/partial pancreatic head resection as mDPPHRt/p; Newcastle–Ottawa scale, NOS.

**Table 2:** Characteristics of the enrolled randomized studies.

Study	Study design	Diagnosis	Study period	Treatment group	Patient number	Sex (M/F)	Age	Follow-up duration (month)
Strate (2005)/Izbicki (1995)	Long-term follow-up of RCT	Chronic pancreatitis	–	Beger	26	–	–	96
				Frey	25	–	–	
Müller (2008)/Büchler (1995)	Long-term follow-up of RCT	Chronic pancreatitis	–	Beger	15	–	–	168
				PPPD	14	–	–	
				Beger	15	–	–	84
				PPPD	14	–	–	
Bachmann (2014)/Izbicki (1995)	Long-term follow-up of RCT	Chronic pancreatitis	–	Beger	22	–	–	192 (range 168–216)
				Frey	23	–	–	
Bachmann (2013)/Izbicki (1998)	Long-term follow-up of RCT	Chronic pancreatitis	–	PPPD	14	–	–	180 (range 168–204)
				Frey	21	–	–	
Klaiber (2016)/Köninger (2008)	Long-term follow-up of RCT	Chronic pancreatitis	–	Beger	18	12/6	59 (45–75)	120
				Berne	22	16/6	58 (23–75)	
Strate (2008)/Izbicki (1998)	Long-term follow-up of RCT	Chronic pancreatitis	1995/1–1997/1	PPPD	23	–	–	84
				Frey	23	–	–	
lzbicki (1995)	RCT	Chronic pancreatitis	1992/1–?	Beger	20	15/5	45.3 ± 8.1	18 (range 6–12)
				Frey	22	16/6	44.1 ± 5.9	
Büchler (1995)	RCT	Chronic pancreatitis	1991/10–1998/8	DPPHR	20	18/2	43 ± 9	6
				PPPD	20	18/2	46 ± 11	
Diener (2017)	RCT, multicenter	Chronic pancreatitis	2009/9–2013/9	DPPHR	115	95/20	52.3 ± 11.1	24
				PD/PPPD	111	86/25	51.5 ± 10.5	
Köninger (2008)	RCT	Chronic pancreatitis	2002/12–2005/1	Beger	32	20/12	48 ± 10	24
				Berne	33	25/8	46 ± 11	
Izbicki (1998)	RCT	Chronic pancreatitis	1995/1–1997/1	Frey	31	25/6	43.1 ± 6.5	24 (range 12–36)
				PPPD	30	26/4	44.6 ± 26	
Farkas (2006)	RCT	Chronic pancreatitis	2002/8–2004/5	DPPHR (Berne)	20	15/5	43 ± 5	12–35
				PPPD	20	15/5	45 ± 8	
Keck (2012)	RCT	Chronic pancreatitis	1992/5–2001/3	PPPD	43	37/6	42.7 (32.6–69.3)	65.6 (range 6–129)
				DPPHR (Beger/Frey)	42	35/7	41.2 (26.8–72.5)	

RCT, randomized controlled trial; Conventional pancreatoduodenectomy or Whipple procedure as PD, pylorus-preserving PD as PPPD, duodenum-preserving pancreatic head resection as DPPHR.

### 3.3. Risk of bias in enrolled studies

None of the RCTs was a low risk or high risk. Supplementary Figure S1A shows the specific evaluation terms of RCT and results. Supplementary Figure S1B shows the summary of the risk of bias in the individual domains. The evaluation of nonrandomized studies was presented as NOS scores, as shown in Table 1. The scores of included nonrandomized studies ranged from 6 to 8. Five studies were excluded due to low quality (NOS score <6).

### 3.4. Meta-analysis of enrolled studies

We defined the treatment options of conventional pancreatoduodenectomy or Whipple procedure as PD, pylorus-preserving PD as PPPD, duodenum-preserving pancreatic head resection as DPPHR, and DPPHR procedure of pancreatic head resection with segmental duodenectomy as PHRSD. Specific DPPHR procedures were named after the reporter's name, such as Beger. Some modifications of DPPHR procedures with total/partial pancreatic head resection were named as mDPPHRt/p. If there was no detailed description of the DPPHR procedure or a mixture of DPPHR, a general DPPHR treatment label was allocated.

**Figure 2:**
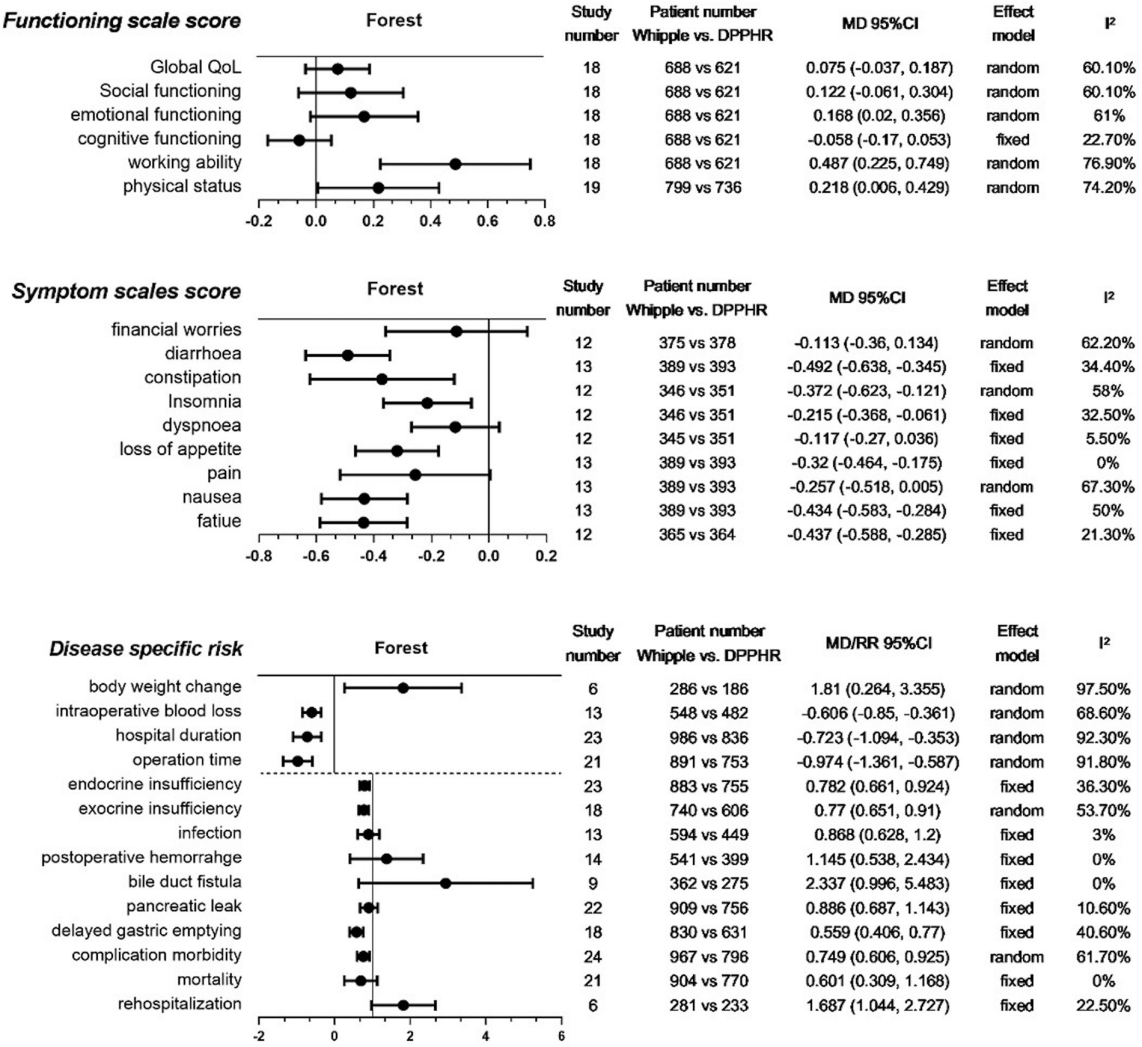
Meta-analysis of collected indexes. Meta-analysis of three categories of indexes: functioning scale scores, symptom scale scores, and disease-specific risks. The last 10 indexes of the third category were analyzed by RR. QoL, quality of life; DPPHR, duodenum-preserving pancreatic head resection; MD, mean difference.

We collected three categories of indexes: disease-specific risks, symptom scale scores, and function scale scores (20). The latter two categories were developed for and mostly reported in the postdischarge assessment of general and pancreatic disease-related QoL (20, 21). Patients who underwent PD and PPPD were classified into the Whipple group, and patients who underwent all DPPHR procedures into the DPPHR group. Each category consisted of several indexes, as shown in Figure 2.

For the function scale score after discharge from the hospital, our meta-analyses showed that there were no significant differences between the two groups in QoL, social function, emotional function, and cognitive function. However, DPPHR had better working ability and physical status (Figure 2).

For the postdischarge symptom scale score, we found that the Whipple group had more severe symptoms than the DPPHR group in diarrhea, constipation, insomnia, loss of appetite, nausea, and fatigue. Financial worries, dyspnea, and pain were comparable between the two groups (Figure 2).

We collected frequently reported disease-specific risk indexes. Body weight change, intraoperative blood loss, length of hospital stay, and operation time were presented as continuous variables. The remaining indexes of this category were presented as binary variables. The meta-analyses revealed that the Whipple group had more body weight loss, intraoperative blood loss, longer hospital stay, longer operation time, and more complications, especially in delayed gastric emptying and endocrine and exocrine insufficiency. Postoperative infection, pancreatic leakage, bile duct fistula, hemorrhage, rehospitalization, and mortality were comparable between the two groups (Figure 2).

The heterogeneity test showed that most meta-analyses of the indexes had low and moderate heterogeneity (*I*^2^ ≤ 50%, 50% < *I*^2^ ≤ 75%). Only working ability, body weight change, length of hospital stay, and operation time had obvious heterogeneity (*I*^2^ > 75%). Each group consisted of several procedures that might contribute to the heterogeneity. These indexes were analyzed in the following network meta-analysis.

### 3.5. Publication bias of meta-analysis

Considering that the meta-analyses of most indexes included more than 10 studies except for three indexes, we constructed funnel plots to assess the publication bias of the studies enrolled in each meta-analysis. By observing the distribution of enrolled studies in the funnel plots, we found that all studies had a rough symmetric funnel shape, indicating no obvious publication bias in each meta-analysis.

### 3.6. Network meta-analysis

Several surgical procedures have been applied to treat pancreatic head benign and low-grade malignant lesions. However, the potential advantages of these surgical procedures remain unclear. We conducted network meta-analyses of indexes that had obvious heterogeneity in meta-analysis and that were mostly reported (nearly half of the enrolled literature in our study) and had significant differences in the meta-analysis (Table 3). Only a single procedure was compared in this part.

**Table 3:** Subgroups of network meta-analysis.

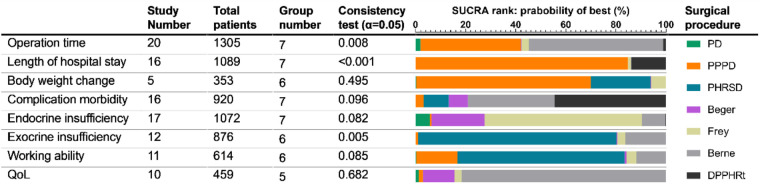

#### 3.6.1. Operation time

**Figure 3:**
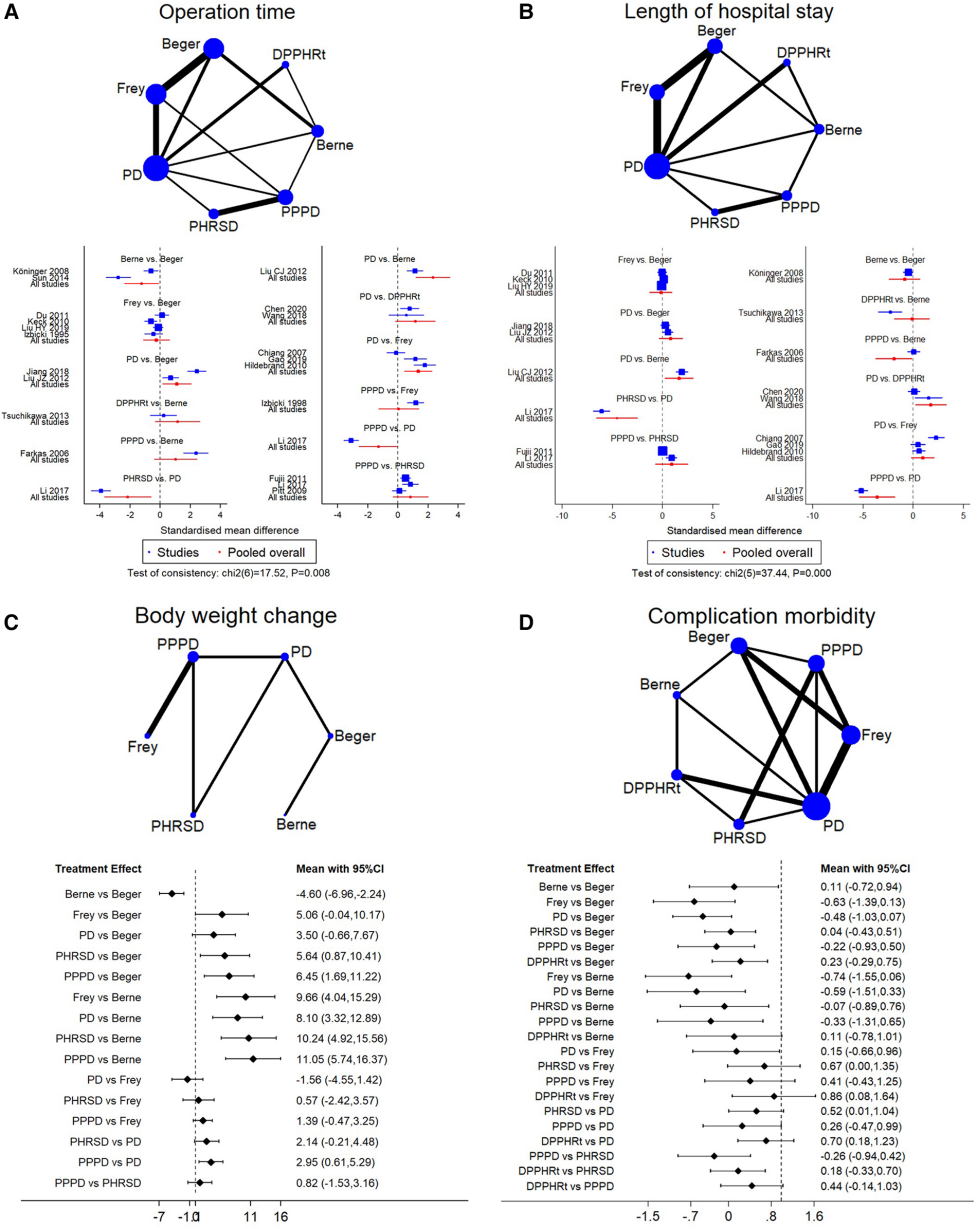
Network meta-analysis. (**A**) Operation time, (**B**) length of hospital stay, (**C**) body weight change, and (**D**) complication morbidity.

We identified 20 studies including seven procedures that were eligible for the analysis. Owing to the consistency test showing *p* = 0.008, only 12 direct comparisons of 21 comparisons in the network analysis were kept. The surface under the cumulative ranking curve (SUCRA) was used to provide the probability of the best treatment. The result showed that Berne (55.9%), PPPD (36.8%), and Frey (3.3%) procedures had the largest probability of the shortest operation time (Table 3). The forest plot also supported that DPPHR procedures were shorter than PD (Figure 3A).

#### 3.6.2. Length of hospital stay

Sixteen studies including seven procedures were analyzed in this part. The consistency test showed *p* < 0.001. The eligible 11 direct comparisons in the network analysis found that PPPD, Berne, DPPHRt, and PHRSD were shorter than PD and PPPD was shorter than Berne in terms of the length of hospital stay (Figure 3B). The SUCRA rank result also had a similar trend that PPPD (84.8%), DPPHRt (13.9%), and Frey (1.2%) had the most possible and shortest hospital stay than other procedures (Table 3).

#### 3.6.3. Body weight change

Five studies including six procedures were analyzed in this part. The consistency test showed *p* = 0.495. The network meta-analysis results found that eight out of 15 comparisons had statistically significant difference. PHRSD [MD: 5.64 (0.87, 10.41)] and PPPD [MD: 6.45 (1.69, 11.22)] lost more weight than Beger. PPPD [MD: 11.05 (5.74, 16.37)], PHRSD [MD: 10.24 (4.92, 15.56)], Beger [MD: −4.60 (−6.96, −2.24)], and Frey [MD: 9.66 (4.04, 15.29)] lost more body weight than Berne. However, PPPD had no difference compared to PHRSD [MD: 0.82 (−1.53, 3.16)] (Figure 3C). The SUCRA rank result indicated that PPPD (69.7%), PHRSD (23.9%), and Frey (6%) had the largest probability of remarkably losing body weight after surgery (Table 3).

#### 3.6.4. Complication morbidity

Sixteen studies including seven procedures were analyzed in this part. The consistency test showed *p* = 0.096. The network meta-analysis results found that the advantage of DPPHRt, PHRSD, Frey, Berne, and Beger in complication morbidity gradually decreased in order. In contrast, the comparison between PPPD/PD and DPPHRt/PHRSD needs further validation (Figure 3D). The SUCRA rank result indicated that DPPHRt (44.5%), Berne (34.5%), and PHRSD (10%) had the largest probability of least complication morbidity (Table 3).

#### 3.6.5. Endocrine insufficiency

**Figure 4:**
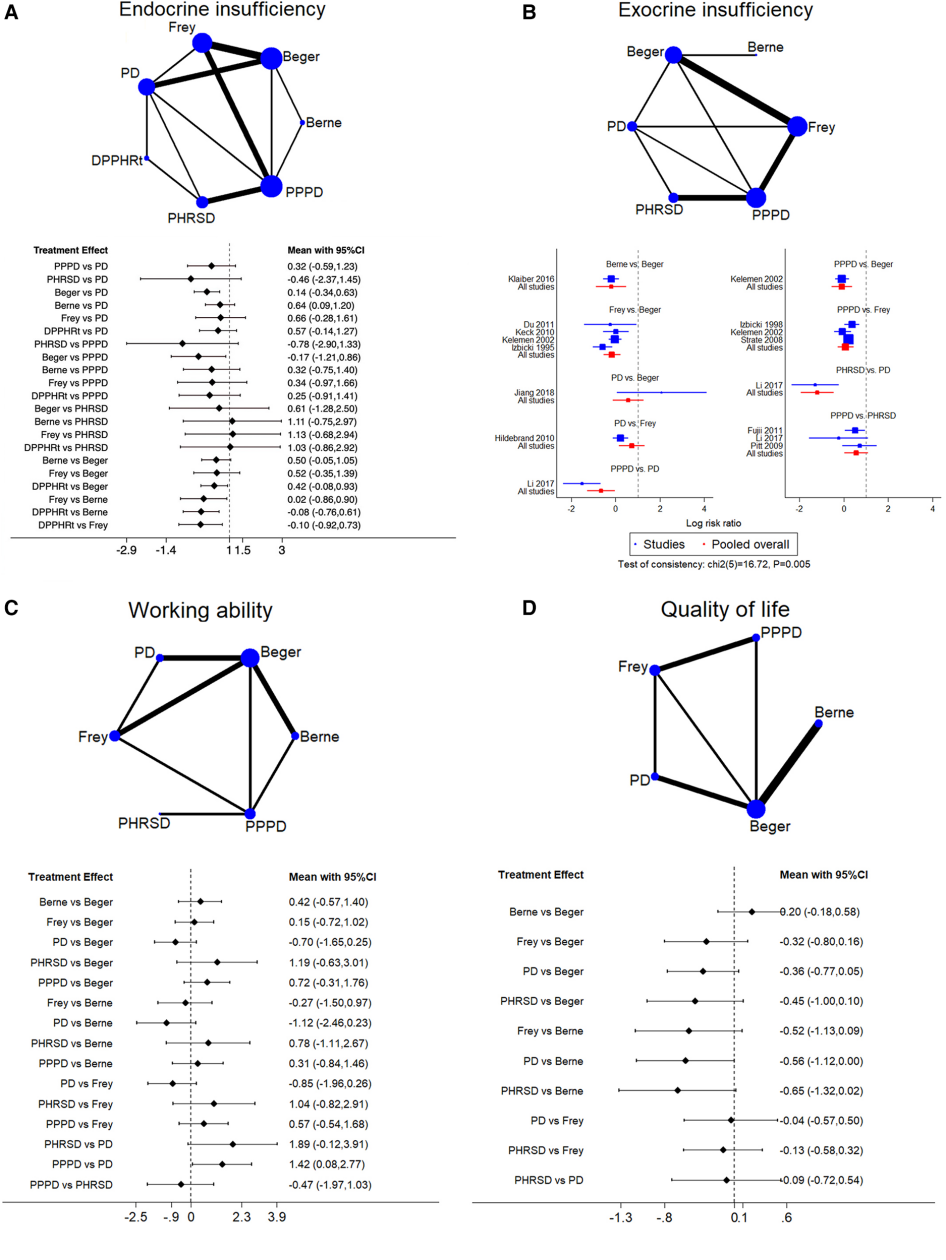
Network meta-analysis. (**A**) Endocrine insufficiency, (**B**) exocrine insufficiency, (**C**) working ability, and (**D**) qualify of life.

Seventeen studies including seven procedures were analyzed in this part. The consistency test showed *p* = 0.082. Beger had less endocrine insufficiency than PPPD [RR: −0.17 (−1.21, 0.86)] and PD [RR: 0.14 (−0.34, 0.63)]. DPPHRt, Frey, and Berne have relative advantages over each other in order (Figure 4A). Frey (62.7%), Beger (21.3%), and Berne (9.3%) had the largest probability of least endocrine insufficiency morbidity (Table 3).

#### 3.6.6. Exocrine insufficiency

Twelve studies including six procedures were analyzed in this part. The consistency test showed *p* = 0.005. The network meta-analysis showed that Berne, Frey, and PPPD had less morbidity than Beger. PPPD and PHRSD had less morbidity than PD (Figure 4B). PHRSD (79.2%), Berne (16.3%), and Frey (3.2%) had the largest probability of least exocrine insufficiency morbidity (Table 3).

#### 3.6.7. Working ability

Eleven studies including six procedures were analyzed in this part. The consistency test showed *p* = 0.085. The network meta-analysis showed that only one comparison had statistical significance. PPPD was better than PD [SMD: 1.42 (0.08, 2.77)] (Figure 4C). PHRSD (66.7%), PPPD (16.5%), and Berne (11.8%) had the most possible best working ability after discharge than other procedures (Table 3).

#### 3.6.8. Quality of life

Ten studies including five procedures were analyzed in this part. The consistency test showed *p* = 0.682. The network meta-analysis found that all comparisons had no statistical significance, which might mean that all procedures had a similar effect on improving the postoperative QoL of patients (Figure 4D). However, the SUCRA rank still showed a potential trend that Berne (81.1%), Berger (12.7%), and Frey (2.7%) had a larger possibility of improving QoL than PPPD (1.7%) and PD (1.2%) (Table 3).

### 3.7. Publication bias of network meta-analysis

All network meta-analysis subgroups had at least six direct comparisons. We constructed funnel plots to assess publication bias in each subgroup. We observed a rough symmetric funnel shape, which indicated that there was no obvious publication bias in each subgroup (Supplementary Figure S2).

## 4. Discussion

To deeply investigate the surgical treatment of pancreatic head benign and low-grade malignant lesions, this study contained accessible literature including large amounts of retrospective and prospective cohort studies, RCTs, and long-term follow-ups of RCT from six databases and enrolled many more legal literature works than any previous meta-analysis publications (11, 22–26). We provided multidimensional comprehensive comparisons among surgical procedures.

Through meta-analyses, we found that PD/PPPD and DPPHR had equal ability to improve postoperative QoL, pain relief, and other functions. This consisted of the latest RCT (27) and previous meta-analysis of RCTs (22). Different literature works reported outcomes in short-term or long-term follow-ups, comparing PD/PPPD with DPPHR. In contrast, our synthesis had moderate heterogeneity for QoL (60.0%) and symptom of pain (67.3%). Pooled data also showed that these two interventions had no differences in improving short-term and long-term QoL and pain relief (22, 26). Subgroup meta-analysis of RCTs or observational studies supported that PD/PPPD and DPPHR had no difference in postoperative pain relief (25). All evidence indicated that both interventions could have equal effects on improving QoL and pain relief in short-term and long-term discharge days.

Each intervention may have different disadvantages and advantages. The advantages of DPPHR included improved working ability, physical status, and body weight compared with PD/PPPD. PD/PPPD had more severe symptoms and more disease-specific risks such as more intraoperative blood loss, longer length of hospital stay, longer operation time, and more complications. Although we disagreed with Strate et al. (28) and Bachmann et al. (12) in terms of working ability and physical status, our results had the same tendency as Zhao et al. (26) with meta-analysis of RCTs and Möbius et al. (29) with a long-term follow-up of a prospective cohort study. In addition, the short-term and long-term effects of these interventions were consistent (26). For the comparison of symptom scale scores and disease-specific risks, our pooled results were generally consistent with most results of previous meta-analyses (11, 25, 26). Endocrine and exocrine insufficiency morbidity after surgery have different definitions in various literature works. More uniformed oral glucose tolerance test (OGTT) was tested for endocrine insufficiency, and fecal elastase concentration measurement was used for exocrine function examination (29–34); however, other methods were mentioned (35–39) or could not find descriptions in some articles. These reasons together with article enrollment discrepancy may cause inconsistency of pooled results compared to former partial meta-analyses (24–26).

Owing to good quantification methods defined by pioneer studies, pooled results of the function and symptom scales did not have obvious heterogeneity. Other indexes like endocrine and exocrine insufficiency reports in the literature may have different definitions or cutoff values or classifications. Our study enrolled 44 pieces of literature, but these studies had various baseline characteristic contents and most had a small sample size. What is more, data were presented in an inconsistent format. These would contribute to heterogeneity. However, we observed that only four of 29 indexes had obvious heterogeneity, 10 of 29 indexes had moderate heterogeneity, and more than half had low heterogeneity. Although not all pooled results were consistent with the previous meta-analyses, we included all studies of their enrollment and more legal pieces of literature to get more exact and representative results.

Another heterogeneity source found that we included multiple procedures in each Whipple/DPPHR group and each procedure had individual properties. In this study, we enrolled Beger, Frey, Berne, mDPPHRt, mDPPHRp, and PHRSD in the DPPHR group. Since Beger et al. presented DPPHR, many modifications have been reported. We made a list and summary of them in Supplementary Table S1. We could see that excision extension differed from specific consideration. These procedures involved resection of the duodenum, partial/total pancreatic head tissue, pancreatic body/tail tissue, common bile duct, and pancreatoduodenal artery arch with the corresponding anastomosis. As time went by, new surgical techniques emerged. Laparoscopy (Supplementary Table S1) and robot-assisted (also called minimally invasive) (39, 40) pancreas surgeries have been done, but their strengths and weaknesses needed further investigation (41–44). In addition, Wang et al. (45) recently designed and performed one novel surgical procedure (Duodenum-CBD-Oddi's Sphincter-preserving Pancreatic Head *en bloc* Total Resection, DCOPPHTR, Wang's procedure) for CP and CP with pancreatolithiasis, while Wang's procedure was also extended to treat benign or low-grade malignant tumors of the pancreatic head. They performed a series of cases and made 9-year long-term follow-ups for all patients who underwent Wang's procedure. It confirmed that Wang's procedure broke through DPPHR's principles, while the efficacy and safety were excellent. Open and laparoscopic mDPPHRt were achieved in our and other centers (Supplementary Table S1) due to a comprehensive understanding of pancreatic anatomy (46, 47), while the investigation of the differences between DPPHR procedures was still deficient (13, 14).

We conducted eight subgroups of network meta-analyses of indexes holding obvious heterogeneity and most frequently reported in our meta-analyses. We found that PPPD/PD and DPPHR procedures had distinct strengths. DPPHR procedures had a larger probability of best performance in seven of eight analyzed indexes. PD showed the lowest probability of getting the best outcomes, but it could potentially remove pre-/minimal malignant lesions. Although SUCRA rank gave us an intuitional judgment of the best choice, pooled results presented in the forest plot found that not all procedures significantly differed from the others.

Network meta-analysis detected an obvious inconsistency in operation time, length of hospital stays, and exocrine insufficiency, which may also indicate heterogeneity among comparisons derived from different studies. The previous meta-analysis also detected obvious heterogeneity in the pooled results of operation time (11, 23, 24). More uniform objective research designed for these indexes in single-procedure comparison could bring us more solid evidence. Meta-analysis and network meta-analysis had the same outcome that PD/PPPD and DPPHR were equal in improving QoL, while network analysis overturned the result of meta-analysis in working ability. It stressed the necessity of exploring heterogeneity and equality in the working ability improvement of interventions (12, 28). Generally, we provided delicate comparisons for clinical reference.

Both parts of our analysis had an unapparent publication bias. Data in different formats from the enrolled literature were collected and converted, which may add uncertainty to the results. However, more usable data could provide more confirmed and elaborate subgroup analysis. Different studies had different definitions for one parameter, such as loss of body weight and weight gain. This contributed to reducing the availability of limited published data.

## 5. Conclusion

Our analysis found that DPPHR and PD/PPPD had equality in improving QoL and pain relief, while PD/PPPD had more severe symptoms and more complications after surgery. PD, PPPD, and DPPHR procedures had different strengths in treating pancreatic head benign and low-grade malignant lesions. Surgeons should pay more attention to identifying characteristics of procedures and patients' clinical features to provide precision treatment with individualized surgical procedures.

## Data Availability

The original contributions presented in the study are included in the article/Supplementary Material; further inquiries can be directed to the corresponding author/.
