# Supplementary material for: Duodenum-preserving pancreatic head resection compared to pancreaticoduodenectomy: A systematic review and network meta-analysis of surgical outcomes
Source: Front Surg. 2023 Mar 29;10:1107613. doi: 10.3389/fsurg.2023.1107613 (PMC10090510; doi:10.3389/fsurg.2023.1107613)
Supplement: Supplementary file 1 [file Datasheet1.docx]

Supplementary Material

Duodenum-preserving pancreatic head resection is superior for pancreatic head benign or low-malignant lesions: a systematic review and network meta-analysis

Shixiang Guo, Qiang Zhou, Jiali Yang, Junyu Tao, Junfeng Zhang, Huaizhi Wang^*^

*** Correspondence:** Huaizhi Wang: [whuaizhi@gmail.com](mailto:whuaizhi@gmail.com)

# Supplementary Figures

## Supplementary Figure S1. Risk of bias assessment of RCTs


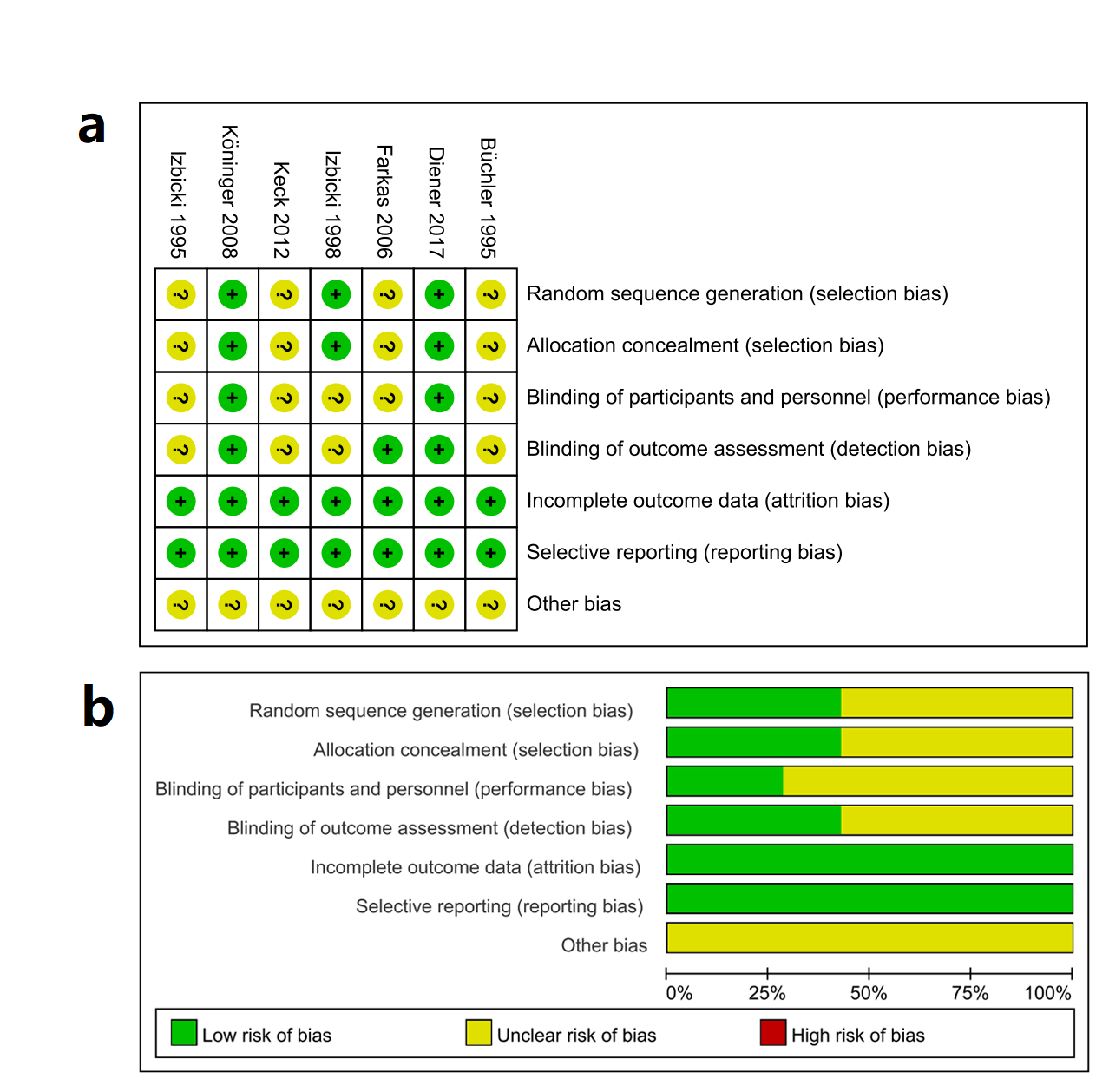


## Supplementary Figure S2. Funnel plots of network meta-analysis


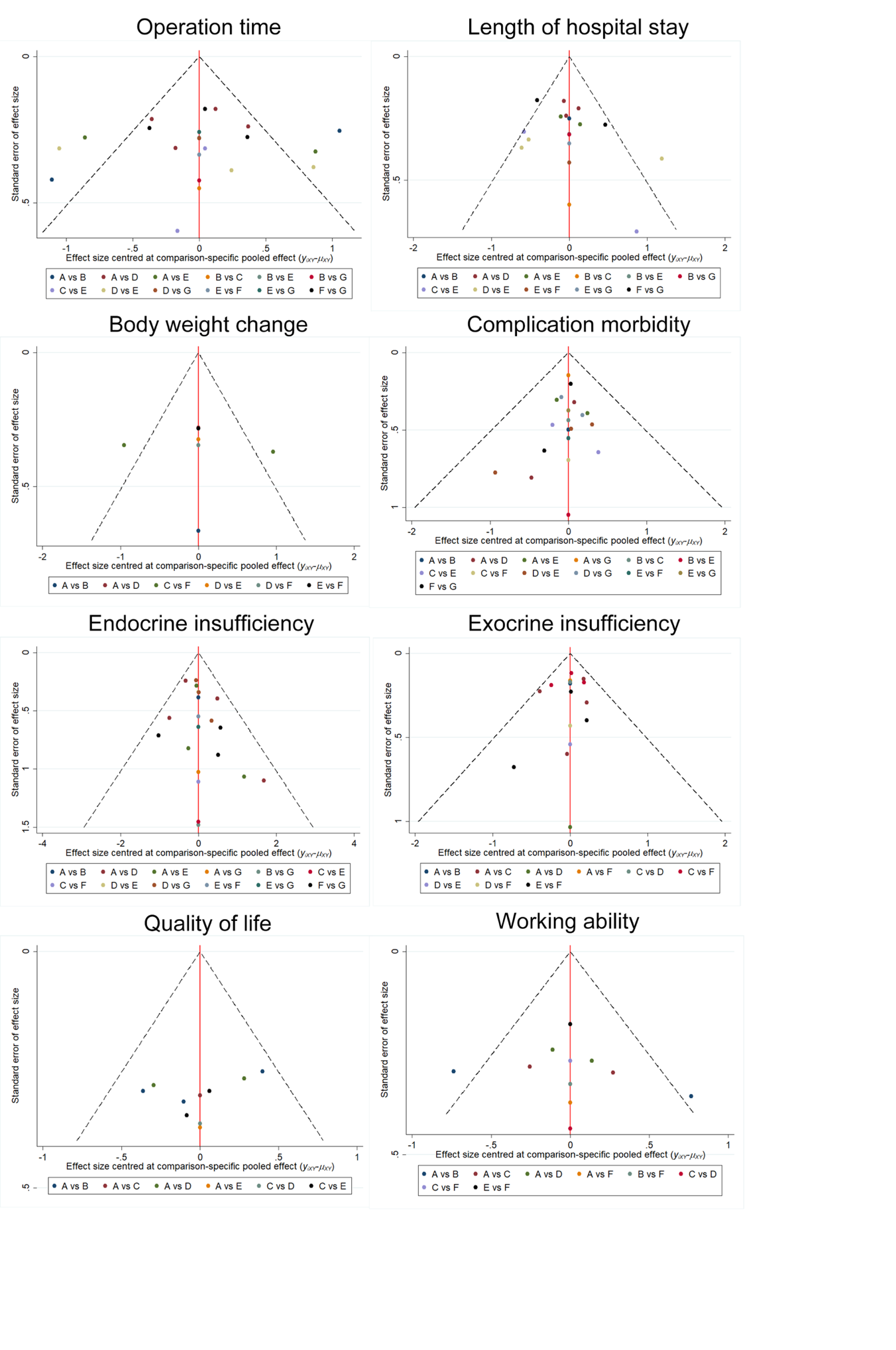


# Table S1. characteristics duodenum-preserving pancreatic head resection (DPPHR) procedures

| Name of surgical procedures | |  | Characteristics | |
| --- | --- | --- | --- | --- |
| DPPHR procedure | **Abbreviation** |  | **Resection** | **Anastomosis** |
| Beger procedure of DPPHR | Beger | | Pancreas is divided above the portal vein and the inflammatory mass is removed from the pancreatic head, leaving a 5- to 8-mm rim of tissue adjacent to the duodenal wall and posterior wall. | Roux-en-Y end-to-side pancreaticojejunostomy (PJS);  A side-to-side jejunum anastomosis to the excavated pancreatic head |
| Frey procedure of DPPHR | Frey | | Limited excavation of the pancreatic head; combined with a longitudinal V shape resection down to pancreatic duct in pancreatic body. | Side-to-side PJS |
| Berne modification of DPPHR | Berne | | The anterior capsule of the pancreatic head is incised; the enlarged inflammatory mass is almost entirely removed leaving only a thin rim of pancreatic head tissue; common bile duct (CBD) is frequently incised. | Side-to-side PJS |
| Modified DPPHR  (Tsuchikawa 2013(1)) | DPPHRt | | All the parenchyma around the pancreatic head, including the groove area were removed, other steps are similar to Beger procedure. | Roux-en-Y PJS |
| Duodenum-preserving pancreatic head resection  (Horiguchi 2010(2)) | DPPHRp | | Remain the pancreatic tissue of the right-latero-dorsal side of the bile duct to avoid injury of the PSPDA; 0ther steps similar to Beger procedure. | Roux-en-Y end-to-side PJS |
| Laparoscopic Duodenum-Preserving Total Pancreatic Head Resection  (Cao 2019(3), Chen 2020(4)) | LDPPHRt | | Laparoscopic assistant surgery; pancreas was cut at neck after establishing post pancreatic tunnel; SAPDA was resected; CBD was preserved; the main pancreatic duct to the ampulla of Vater was dissected; the pancreatic head (or with uncinate process) were completely removed. | Roux-en-Y end-to-side  PJS, or pancreaticogastrostomy (PGS) |
| Pancreatic head resection with segmental duodenectomy  (Nakao 1994/2007(5)) | PHRSD | | The oral side of the duodenum is divided at 5 to 7 cm from the pyloric ring. The anal side of duodenum is divided at the point of AIPDA ligation; pancreatic neck is divided; pancreatic segmental CBD is resected; pancreatic head is totally removed. | Type A: end-to-side PGS, duodenoduodenostomy, and choledochoduodenostomy;  Type B: Roux-en-Y end-to-side PJS, duodenoduodenostomy, and cholecochojejunostomy. |
| Proximal parenchymal pancreatectomy  (Umemoto 2019(6)) | PPP  (DPPHRt) | | Duodenum and CBD preserving; preserving posterior vascular arcade; complete resection of the pancreatic head parenchyma, including the groove area. | Roux-en-Y end-to-side PJS |
| Imaizumi modification of the Beger procedure (Hatori 2010(7)) | Imaizumi (DPPHRp) | | Duodenum preserving; ligating PDA anterior arch, preserving PDA posterior arch; pancreatic segment of CBD is removed; subtotal pancreatic head resection (pancreatic head is divided at a distance of 4–5 mm from the wall of the duodenum toward the papilla of Vater). | Roux-en-Y end-to-side PJS ;  end-to-side choledochoduodenostomy or choledochojejunostomy |
| Duodenum-Preserving and Bile Duct-Preserving Total Pancreatic Head Resection  (Takada 1993/2004(8), Nagakawa 1997(9)) | DPTPHR (DPPHRt) | | Ligating PDA anterior arch, preserving PDA posterior arch and mesoduodenal vessels; preserving duodenum, CBD, duodenal papilla, and Oddi’s sphincter; pancreatic head totally resected; proximal pancreatic duct is cut with sufficient length to perform the end-to-end pancreatic anastomosis. | End-to-end pancreatic duct-to-duct anastomosis |
| A modification of both the Beger and the Frey procedures  (Gloor 2001(10)) | Gloor (DPPHRp) | | the anterior capsule of the pancreatic head is incised and the enlarged pancreatic head is excised almost in its entirety, leaving behind only a thin bridge of pancreatic tissue between head and body and an opened common bile and pancreatic duct. | end-to-side PJS or Frey’s anastomosis |
| Laparoscopic Duodenum-Preserving Pancreatic Head Resection  (Chen S. 2020(11), Hong 2021(12), Cai 2021(13)) | LDPPHR | | Laparoscopic surgery; indocyanine green (ICG) to visualize and preserve CBD and the vessels supplying the duodenum and CBD; other steps are similar to Beger procedure. | Pancreatic duct end-to-end anastomosis, or PJS |
| 3 types of modified duodenum-preserving pancreatic head resection (Pedrazzoli 2011(14)) | Type 1: Beger  Type 2: mBeger  Type3: DPPHRt | | Type 2: all the pancreatic tissue on the inner surface of the duodenum inferior to Vater’s papilla is removed with preserving inferior pancreaticoduodenal vessels;  Type 3: CBD is skeletonized over its entire circumference until Vater’s papilla, the pancreatic head is entirely removed. | Roux-en-Y end-to-side PJS |
| Robot-assisted Duodenum-preserving Pancreatic Head Resection (Peng 2012(15)) | RDPPHR | | Robot-assisted surgery; similar to Beger procedure. | End-to-side PGS |
| Extended retrograde drainage of the pancreatic duct (modified Puestow procedure)  (Isaji 2010(16)) | Partington procedure | | Open pancreatic duct from the distal most portion of the tail to the right of the mesenteric vessels. | Roux-en-Y side-to-side PJS |
| Longitudinal V-Shaped Excision of the Ventral Pancreas (Izbicki 1998(17)) | Hamburg (DPPHRp) | | Starting from the upper and lower edges of the head, the ventral pancreatic aspect was longitudinally excised, with the tip of the excised deep in the dorsal part of the pancreas. | Roux-en-Y end-to-side PJS |
| Duodenum-CBD-Oddi's Sphincter-preserving Pancreatic Head en bloc Total Resection (Guo 2021(18)) | DCOPPHTR (DPPHRt) | | Pancreatic neck was cut after establishing post-pancreatic tunnel; anterior and posterior arches were preserved; the pancreatic duct to the ampulla of Vater was dissected, CBD and Oddi’s sphincter were preserved; pancreatic head was dissected along the outside of capsule and totally removed. | Roux-en-Y end-to-side PJS |

**References of Table S1**

1. Tsuchikawa T, Hirano S, Tanaka E, Kato K, Matsumoto J, Nakamura T, et al. Modified Duodenum-Preserving Pancreas Head Resection for Low-Grade Malignant Lesion in the Pancreatic Head. *Pancreatology : official journal of the International Association of Pancreatology (IAP) [et al]* (2013) 13(2):170-4. Epub 2013/04/09. doi: 10.1016/j.pan.2012.12.001.

2. Horiguchi A, Miyakawa S, Ishihara S, Ito M, Asano Y, Furusawa K, et al. Surgical Design and Outcome of Duodenum-Preserving Pancreatic Head Resection for Benign or Low-Grade Malignant Tumors. *J Hepatobiliary Pancreat Sci* (2010) 17(6):792-7. Epub 2009/11/07. doi: 10.1007/s00534-009-0221-4.

3. Cao J, Li GL, Wei JX, Yang WB, Shang CZ, Chen YJ, et al. Laparoscopic Duodenum-Preserving Total Pancreatic Head Resection: A Novel Surgical Approach for Benign or Low-Grade Malignant Tumors. *Surg Endosc* (2019) 33(2):633-8. Epub 2018/11/21. doi: 10.1007/s00464-018-6488-2.

4. Chen X, Chen W, Zhang Y, An Y, Zhang X. Short-Term Outcomes of Laparoscopic Duodenum-Preserving Total Pancreatic Head Resection Compared with Laparoscopic Pancreaticoduodenectomy for the Management of Pancreatic-Head Benign or Low-Grade Malignant Lesions. *Medical science monitor : international medical journal of experimental and clinical research* (2020) 26:e927248. Epub 2020/09/17. doi: 10.12659/msm.927248.

5. Nakao A, Fernández-Cruz L. Pancreatic Head Resection with Segmental Duodenectomy: Safety and Long-Term Results. *Ann Surg* (2007) 246(6):923-8; discussion 9-31. Epub 2007/11/29. doi: 10.1097/SLA.0b013e31815c2a14.

6. Umemoto K, Tsuchikawa T, Nakamura T, Okamura K, Noji T, Asano T, et al. Postoperative Nutritional Benefits of Proximal Parenchymal Pancreatectomy for Low-Grade Malignant Lesions in the Pancreatic Head. *HPB : the official journal of the International Hepato Pancreato Biliary Association* (2019) 21(11):1491-6. Epub 2019/04/10. doi: 10.1016/j.hpb.2019.03.359.

7. Hatori T, Imaizumi T, Harada N, Fukuda A, Suzuki M, Hanyu F, et al. Appraisal of the Imaizumi Modification of the Beger Procedure: The Twmu Experience. *J Hepatobiliary Pancreat Sci* (2010) 17(6):752-7. Epub 2009/09/22. doi: 10.1007/s00534-009-0183-6.

8. Takada T, Yasuda H, Amano H, Yoshida M. A Duodenum-Preserving and Bile Duct-Preserving Total Pancreatic Head Resection with Associated Pancreatic Duct-to-Duct Anastomosis. *Journal of gastrointestinal surgery : official journal of the Society for Surgery of the Alimentary Tract* (2004) 8(2):220-4. Epub 2004/03/24. doi: 10.1016/j.gassur.2003.11.007.

9. Nagakawa T, Ohta T, Kayahara M, Ueno K. Total Resection of the Head of the Pancreas Preserving the Duodenum, Bile Duct, and Papilla with End-to-End Anastomosis of the Pancreatic Duct. *Am J Surg* (1997) 173(3):210-2. Epub 1997/03/01. doi: 10.1016/s0002-9610(97)89594-3.

10. Gloor B, Friess H, Uhl W, Buchler MW. A Modified Technique of the Beger and Frey Procedure in Patients with Chronic Pancreatitis. *Digestive Surgery* (2001) 18(1):21-5. doi: 10.1159/000050092.

11. Chen S, Gao P, Cai H, Cai Y, Wang X, Peng B. Indocyanine Green-Enhanced Fluorescence in Laparoscopic Duodenum-Preserving Pancreatic Head Resection: Technique with Video. *Ann Surg Oncol* (2020) 27(10):3926-7. Epub 2020/04/09. doi: 10.1245/s10434-020-08360-6.

12. Hong D, Cheng J, Wu W, Liu X, Zheng X. How to Perform Total Laparoscopic Duodenum-Preserving Pancreatic Head Resection Safely and Efficiently with Innovative Techniques. *Ann Surg Oncol* (2021) 28(6):3209-16. Epub 2020/10/31. doi: 10.1245/s10434-020-09233-8.

13. Cai Y, Zheng Z, Gao P, Li Y, Peng B. Laparoscopic Duodenum-Preserving Total Pancreatic Head Resection Using Real-Time Indocyanine Green Fluorescence Imaging. *Surg Endosc* (2021) 35(3):1355-61. Epub 2020/03/30. doi: 10.1007/s00464-020-07515-6.

14. Pedrazzoli S, Canton SA, Sperti C. Duodenum-Preserving Versus Pylorus-Preserving Pancreatic Head Resection for Benign and Premalignant Lesions. *J Hepatobiliary Pancreat Sci* (2011) 18(1):94-102. Epub 2010/08/10. doi: 10.1007/s00534-010-0317-x.

15. Peng CH, Shen BY, Deng XX, Zhan Q, Han B, Li HW. Early Experience for the Robotic Duodenum-Preserving Pancreatic Head Resection. *World J Surg* (2012) 36(5):1136-41. Epub 2012/03/15. doi: 10.1007/s00268-012-1503-6.

16. Isaji S. Has the Partington Procedure for Chronic Pancreatitis Become a Thing of the Past? A Review of the Evidence. *Journal of Hepato-Biliary-Pancreatic Sciences* (2010) 17(6):763-9. doi: 10.1007/s00534-009-0181-8.

17. Izbicki JR, Bloechle C, Broering DC, Knoefel WT, Kuechler T, Broelsch CE. Extended Drainage Versus Resection in Surgery for Chronic Pancreatitis: A Prospective Randomized Trial Comparing the Longitudinal Pancreaticojejunostomy Combined with Local Pancreatic Head Excision with the Pylorus-Preserving Pancreatoduodenectomy. *Ann Surg* (1998) 228(6):771-9. Epub 1998/12/22. doi: 10.1097/00000658-199812000-00008.

18. Shixiang G, Huaizhi W. Duodenum-Cbd-Oddi's Sphincter-Preserving Pancreatic Head En Bloc Total Resection (Dcopphtr). *Chin J Oper Proc Gen Surg (Electronic Edition)* (2021) 15(04):374.

# List S1. Studies enrolled in meta-analysis

1. Keck T, Wellner UF, Riediger H, et al. Long-term outcome after 92 duodenum-preserving pancreatic head resections for chronic pancreatitis: comparison of Beger and Frey procedures. J Gastrointest Surg. 2010, 14(3): 549-556.
2. Bellon E, Roswora MD, Melling N, et al. Duodenum-preserving pancreatic head resection: A retrospective analysis of the Hamburg Modification. Surgery. 2019, 165(5): 938-945.
3. Belina F, Fronek J, Ryska M. Duodenopancreatectomy versus duodenum-preserving pancreatic head excision for chronic pancreatitis. Pancreatology. 2005, 5(6): 547-552.
4. Busquets J, Fabregat J, Borobia FG, et al. Organ-preserving surgery for benign lesions and low-grade malignancies of the pancreatic head: a matched case-control study. Surg Today. 2010, 40(2): 125-131.
5. McClaine RJ, Lowy AM, Matthews JB, et al. A comparison of pancreaticoduodenectomy and duodenum-preserving head resection for the treatment of chronic pancreatitis. HPB (Oxford). 2009, 11(8): 677-683.
6. Sun YH, Ding N, Cheng K, et al. Comparative analysis of duodenum-preserving pancreatic head resection and pancreaticoduodenectomy. Chin Med J (Engl). 2020, 133(17): 2112-2113.
7. Witzigmann H, Max D, Uhlmann D, et al. Outcome after duodenum-preserving pancreatic head resection is improved compared with classic Whipple procedure in the treatment of chronic pancreatitis. Surgery. 2003, 134(1): 53-62.
8. Zheng Z, Xiang G, Tan C, et al. Pancreaticoduodenectomy versus duodenum-preserving pancreatic head resection for the treatment of chronic pancreatitis. Pancreas. 2012, 41(1): 147-152.
9. Jiang Y, Jin JB, Zhan Q, et al. Robot-assisted duodenum-preserving pancreatic head resection with pancreaticogastrostomy for benign or premalignant pancreatic head lesions: a single-centre experience. Int J Med Robot. 2018, 14(4): e1903.
10. Li Y, Wu W, Zhang T, et al. Comparison of long-term benefits of organ-preserving pancreatectomy techniques for benign or low-grade malignant tumors at the pancreatic head. Medicine (Baltimore). 2017, 96(51): e9420.
11. Ahn YJ, Kim SW, Park YC, et al. Duodenal-preserving resection of the head of the pancreas and pancreatic head resection with second-portion duodenectomy for benign lesions, low-grade malignancies, and early carcinoma involving the periampullary region. Arch Surg. 2003, 138(2): 162-168; discussion 168.
12. Horiguchi A, Miyakawa S, Ishihara S, et al. Surgical design and outcome of duodenum-preserving pancreatic head resection for benign or low-grade malignant tumors. J Hepatobiliary Pancreat Sci. 2010, 17(6): 792-797.
13. Kelemen D, Horváth OP. Clinical experience with different techniques of pancreatic head resection for chronic pancreatitis. Dig Surg. 2002, 19(1): 28-34; discussion 34-25.
14. Pedrazzoli S, Canton SA, Sperti C. Duodenum-preserving versus pylorus-preserving pancreatic head resection for benign and premalignant lesions. J Hepatobiliary Pancreat Sci. 2011, 18(1): 94-102.
15. Chiang KC, Yeh CN, Hsu JT, et al. Pancreaticoduodenectomy versus Frey's procedure for chronic pancreatitis: preliminary data on outcome and pancreatic function. Surg Today. 2007, 37(11): 961-966.
16. Chen X, Chen W, Zhang Y, et al. Short-Term Outcomes of Laparoscopic Duodenum-Preserving Total Pancreatic Head Resection Compared with Laparoscopic Pancreaticoduodenectomy for the Management of Pancreatic-Head Benign or Low-Grade Malignant Lesions. Med Sci Monit. 2020, 26: e927248.
17. Nakao A, Fernández-Cruz L. Pancreatic head resection with segmental duodenectomy: safety and long-term results. Ann Surg. 2007, 246(6): 923-928; discussion 929-931.
18. Umemoto K, Tsuchikawa T, Nakamura T, et al. Postoperative nutritional benefits of proximal parenchymal pancreatectomy for low-grade malignant lesions in the pancreatic head. HPB (Oxford). 2019, 21(11): 1491-1496.
19. Benzing C, Hau HM, Atanasov G, et al. Surgical therapy of chronic pancreatitis: clinical results and health-related quality of life. Z Gastroenterol. 2018, 56(11): 1354-1364.
20. Hildebrand P, Dudertadt S, Czymek R, et al. DIFFERENT SURGICAL STRATEGIES FOR CHRONIC PANCREATITIS SIGNIFICANTLY IMPROVE LONG-TERM OUTCOME: A COMPARATIVE SINGLE CENTER STUDY. European Journal of Medical Research. 2010, 15(8): 351-356.
21. Chengyi S, Haitao Z. Efficacy of duodenum-preserving pancreatic head resection for chronic pancreatitis with mass in the head of the pancreas. Chin J Dig Surg. 2014, 13(04): 255-258.
22. Jiazhe L, Xinyu H, Hongcheng W, et al. Duodenum-preserving pancreatic head resection versus pancreaticoduodenectomy for chronic pancreatitis. Journal of Surgery Concepts & Practice. 2012, 17(5): 477-480.
23. Changjun L, jinhui Y, Xinmin Y, et al. Compamtive Study of Duodenum-preserving Pancreatic Head Spoon Type Resection and WhippIe Operation for Chronic Pancreatitis With Pancreatic Head Stones Chinese Journal of Modern Operative Surgery. 2012, 16(04): 249-252.
24. Hongying L, Ka L, Nengwen K. Effect of Frey and Beger on pain and quality of life in patients with chronic pancreatitis. Chinese Journal of the Frontiers of Medical Science(Electronic Version). 2019, 11(10): 140-143.
25. Yanan G, Bin W, Zhongtao Z. Comparative study of Frey's and Whipple's operation on pain and quality of life in patients with chronic pancreatitis. Journal of Clinical and Experimental Medicine. 2019, 18(17): 1870-1874.
26. Bingqing D, Yonghua C, Zhenjiang Z, et al. clinical efficacy of Frey and Beger procedures for chronic pancreatitis. Chin J Gen Surg. 2011, (08): 632-634.
27. Kun C, Yonghui S, Hai L, et al. Clinical analysis of long-term effect of duodenum-preserving pancreatic head resection versus pancreaticoduodenectomy: a single center experience. Chinese Journal of Bases and Clinics in General Surgery. 2020, 27(10): 1211-1215.
28. Yuanquan W, Jianguo L. Clinical efficacy of modified Takada procedure duodenum-preserving pancreatic head resection and pancreatoduodenectomy for the treatment of pancreatic head lesions. Chin J Gen Surg. 2018, 33(06): 466-469.
29. Tsuchikawa T, Hirano S, Tanaka E, et al. Modified duodenum-preserving pancreas head resection for low-grade malignant lesion in the pancreatic head. Pancreatology. 2013, 13(2): 170-174.
30. Möbius C, Max D, Uhlmann D, et al. Five-year follow-up of a prospective non-randomised study comparing duodenum-preserving pancreatic head resection with classic Whipple procedure in the treatment of chronic pancreatitis. Langenbecks Arch Surg. 2007, 392(3): 359-364.
31. Fujii T, Kanda M, Kodera Y, et al. Comparison of pancreatic head resection with segmental duodenectomy and pylorus-preserving pancreatoduodenectomy for benign and low-grade malignant neoplasms of the pancreatic head. Pancreas. 2011, 40(8): 1258-1263.
32. Strate T, Taherpour Z, Bloechle C, et al. Long-term follow-up of a randomized trial comparing the beger and frey procedures for patients suffering from chronic pancreatitis. Ann Surg. 2005, 241(4): 591.
33. Müller MW, Friess H, Martin DJ, et al. Long-term follow-up of a randomized clinical trial comparing Beger with pylorus-preserving Whipple procedure for chronic pancreatitis. Br J Surg. 2008, 95(3): 350-356.
34. Bachmann K, Tomkoetter L, Erbes J, et al. Beger and Frey procedures for treatment of chronic pancreatitis: comparison of outcomes at 16-year follow-up. J Am Coll Surg. 2014, 219(2): 208-216.
35. Bachmann K, Tomkoetter L, Kutup A, et al. Is the Whipple procedure harmful for long-term outcome in treatment of chronic pancreatitis? 15-years follow-up comparing the outcome after pylorus-preserving pancreatoduodenectomy and Frey procedure in chronic pancreatitis. Ann Surg. 2013, 258(5): 815-820; discussion 820-811.
36. Klaiber U, Alldinger I, Probst P, et al. Duodenum-preserving pancreatic head resection: 10-year follow-up of a randomized controlled trial comparing the Beger procedure with the Berne modification. Surgery. 2016, 160(1): 127-135.
37. Strate T, Bachmann K, Busch P, et al. Resection vs drainage in treatment of chronic pancreatitis: long-term results of a randomized trial. Gastroenterology. 2008, 134(5): 1406-1411.
38. Izbicki JR, Bloechle C, Knoefel WT, et al. Duodenum-preserving resection of the head of the pancreas in chronic pancreatitis. A prospective, randomized trial. Ann Surg. 1995, 221(4): 350-358.
39. Büchler MW, Friess H, Müller MW, et al. Randomized trial of duodenum-preserving pancreatic head resection versus pylorus-preserving Whipple in chronic pancreatitis. Am J Surg. 1995, 169(1): 65-69; discussion 69-70.
40. Diener MK, Hüttner FJ, Kieser M, et al. Partial pancreatoduodenectomy versus duodenum-preserving pancreatic head resection in chronic pancreatitis: the multicentre, randomised, controlled, double-blind ChroPac trial. Lancet. 2017, 390(10099): 1027-1037.
41. Köninger J, Seiler CM, Sauerland S, et al. Duodenum-preserving pancreatic head resection--a randomized controlled trial comparing the original Beger procedure with the Berne modification (ISRCTN No. 50638764). Surgery. 2008, 143(4): 490-498.
42. Izbicki JR, Bloechle C, Broering DC, et al. Extended drainage versus resection in surgery for chronic pancreatitis: a prospective randomized trial comparing the longitudinal pancreaticojejunostomy combined with local pancreatic head excision with the pylorus-preserving pancreatoduodenectomy. Ann Surg. 1998, 228(6): 771-779.
43. Farkas G, Leindler L, Daróczi M, et al. Prospective randomised comparison of organ-preserving pancreatic head resection with pylorus-preserving pancreaticoduodenectomy. Langenbecks Arch Surg. 2006, 391(4): 338-342.
44. Keck T, Adam U, Makowiec F, et al. Short- and long-term results of duodenum preservation versus resection for the management of chronic pancreatitis: a prospective, randomized study. Surgery. 2012, 152(3 Suppl 1): S95-s102.
